# A Transcriptome Analysis Identifies Biological Pathways and Candidate Genes for Feed Efficiency in DLY Pigs

**DOI:** 10.3390/genes10090725

**Published:** 2019-09-18

**Authors:** Xingwang Wang, Shaoyun Li, Jie Wu, Rongrong Ding, Jianping Quan, Enqin Zheng, Jie Yang, Zhenfang Wu

**Affiliations:** College of Animal Science and National Engineering Research Center for Breeding Swine Industry, South China Agricultural University, Guangzhou 510642, Guangdong, China; wangxw@stu.scau.edu.cn (X.W.); lishaoyun91@163.com (S.L.); wujiezi163@163.com (J.W.); drr_scau@foxmail.com (R.D.); qjp_scau@outlook.com (J.Q.); eqzheng@scau.edu.cn (E.Z.)

**Keywords:** DLY pig, feed efficiency, small intestines, *SLC2A2*

## Abstract

Feed cost accounts for approximately 65–75% of overall commercial pork production costs. Therefore, improving the feed efficiency of pig production is important. In this study, 12 individuals with either extremely high (HE) or low (LE) feed efficiency were selected from 225 Duroc × (Landrace × Yorkshire) (DLY) pigs. After the pigs were slaughtered, we collected small intestine mucosal tissue. Next, RNA sequencing (RNA-seq) analysis was used to reveal the presence and quantity of genes expressed between these extremely HE- and LE-groups. We found 433 significantly differentially expressed genes (DEGs) between the HE- and LE-groups. Of these, 389 and 44 DEGs were upregulated and downregulated in the HE-group, respectively. An enrichment analysis showed that the DEGs were mainly enriched in functions related to apical plasma membrane composition, transporter activity, transport process and hormone regulation of digestion and absorption. Protein network interaction and gene function analyses revealed that *SLC2A2* was an important candidate gene for FE in pigs, which may give us a deeper understanding of the mechanism of feed efficiency. Furthermore, some significant DEGs identified in the current study could be incorporated into artificial selection programs for increased feeding efficiency in pigs.

## 1. Introduction

The efficient usage of resources is a major concern in agri-food production. Efficient use of feed resources has become a clear challenge for the pork industry as feed costs continue to be the largest variable expenditure [1]. Feed cost accounts for approximately 65–75% of overall commercial pork production costs and 75% of this amount is fed in the grow-finish phase of production [2]. Thus, improving feed efficiency (FE) is an important approach for reducing pig production expenses. Additionally, improving FE implies a reduction in the amount of minerals, heavy metal and greenhouse gas excreted per kg of meat produced [3], thus reducing the environmental impact of pig production systems. China is the leading pork producer worldwide, and the dominant commercial pig breed is represented by Duroc × (Landrace × Yorkshire) (DLY) crossbred pigs [4,5]. Thus, solving the problem of DLY feed efficiency is critical for improving the overall efficiency of the pig industry.

Feed efficiency is typically measured as either the feed conversion ratio (FCR) or residual feed intake (RFI) [6]. As a complex trait, FE is determined by both genetics and environmental factors, including diet and rearing conditions [7]. Animal-intrinsic factors play an important role in FE improvement. This is reflected by the considerable FE improvements observed following the implementation of genetic information in breeding programs over the last decades [8]. Of the substantial individual differences identified in FE, approximately 30% are explained by genetics [1,9,10]. Previous studies indicated that the heritability of FCR is 0.13–0.31, while RFI is 0.14–0.40 [11,12,13], and a strong correlation exists between FCR and RFI (R equals 0.76-0.99) [11]. Thus, genetic improvement may enhance FE.

To date, DNA variants that play a role in feed efficiency traits have been detected by genome-wide association studies (GWAS) in pigs. Based on their genomic location relative to these SNPs, putative gene candidates for feed efficiency traits have been suggested. Quantitative trait loci (QTL) mapping studies revealed several important QTL regions for FE-related traits [14,15,16,17]. Furthermore, GWAS identified candidate genes that may affect pig FE, including *PLCB1* (*phospholipase C beta 1*), *GNAS* (*GNAS complex locus*) and *PRL* (*prolactin*), which are involved in hormone metabolism and digestive enzyme secretion processes [18,19].

However, because of multiple biological functions of a single gene, regions and genes identified by GWAS cannot further explain how they affect phenotypes in biological processes and gene network regulation. FE candidate genes pertain to numerous biological processes [14,20], suggesting that the biological strategies recruited for improving feed efficiency are diverse. To examine the variety of functional pathways underlying interindividual differences in feed efficiency, an increasing number of studies have addressed the transcriptomes and miRNA profiles of tissues and organs involved in energy homeostasis and energy demand in pigs [21,22,23,24,25]. FE-related transcriptome research focuses mainly in five tissues: liver [21,23,25], adipose [21,23,26], muscle [23,24], blood [27], and intestine [28,29]. In these previous studies, transcriptome was performed in intestinal tissue to study feed efficiency, a Landrace [29], and a three-way crossbred pig population (Pietrain × (Landrace × Yorkshire)) [28]. However, none of transcriptome analysis in intestines was used to research the mechanism of feed efficiency in DLY pigs, which dominate the global porcine industry.

This study analyzed the transcriptomic profiles of small intestine mucosal tissues from DLY commercial pigs with either high or low feed efficiency to identify important candidate genes and signaling pathways that potentially affect pig feed efficiency, and to enrich the candidate genes for feed efficiency in different pig breeds. This research not only promotes understanding of feed efficiency mechanisms but also offers new insights into the genetic improvement of pig feed efficiency, which could benefit the pig industry.

## 2. Materials and Methods

### 2.1. Ethics Statement

All experimental procedures met the guidelines of the Animal Care and Use Committee of the South China Agricultural University (SCAU) (Guangzhou, China). The Animal Care and Use Committee of the SCAU approved all animal experiments described in this study. Every effort was taken to minimize animal suffering.

### 2.2. Animals and Tissues

In this experiment, 225 female DLY pigs were provided by Guangdong Wen’s Foodstuffs Group Co., Ltd. (Yunfu, China). The pigs were housed in an environmentally controlled room and given feed and water ad libitum throughout the experiment. Pedigree information was available for all pigs. The phenotypic data of all 225 pigs were measured using the Osborne FIRE Pig Performance Testing System (Osborne, KS, USA) as previously described [18]. Animals participated in the trial from around 30 kg of body weight (BW) until around 100 kg BW. The duration of the trial was approximately 12 weeks. Each animal was labeled with a unique, electric identification tag on its ear that could be captured by the automatic feeder. The time, duration, feed consumption, and BW of each pig were recorded at every visit to the feeder. The FCR was calculated for individuals during the trial period. RFI was computed using methods similar to those used by Cai et al [9]. In the model, predicted daily feed intake (DFI) was estimated using linear regression of DFI on mid-test metabolic BW (MWT), average daily gain (ADG) from 30–100 kg, and back fat (BF). MWT was equal to ((BW at on-test + BW at off-test)/2)^0.75^. The FCR and RFI values of all individuals were ordered. Six pigs with extremely high FE and six with extremely low FE were selected from the 225 pigs, and they represent the HE-group and the LE-group, respectively. These selected pigs were slaughtered after the end of the experiment, and their small intestine mucosa tissues were collected immediately. Then, these samples were snap frozen in liquid nitrogen and stored at −80 °C.

### 2.3. RNA Preparation

Approximately 1 mg of small intestine mucosal tissue was used for RNA extraction. The tissue was resuspended in 1 ml of TRIzol (Invitrogen, Carlsbad, CA, USA) and homogenized by a vortex. RNA extraction continued following the standard TRIzol protocol (Invitrogen, Carlsbad, CA, USA). RNA integrity was checked using 1% agarose gel electrophoresis. The RNA was quantified using the NanoDrop ND-2000 spectrophotometer (Thermo Scientific NanoDrop, Wilmington, NC, USA).

### 2.4. RNA Sequencing

The Agilent 2100 Bioanalyzer device (Agilent Technologies, Santa Clara, CA, USA) was used to assess the integrity of the RNA. The RNA integrity value (RIN) of the samples ranged between 7.1–9.9. Paired-end libraries with fragments of 500 bp were prepared, using the TruSeq RNA Library Prep Kit v2 (Illumina, San Diego, CA, USA) according to the manufacturer’s instructions, with 1 µg of RNA. The libraries were sequenced on an Illumina HiSeq4000 platform to obtain PE150 reads.

### 2.5. Quality Control, Mapping, and Quantification

The raw reads were quality controlled before mapping. The FastQC [30] software was used to assess the raw sequencing data quality. High-quality clean reads were obtained by trimming the adapter sequences and removing the reads that adapter contamination is greater than 5 bp, the reads Q20 ratio does not reach 85%, the reads with length less than 149 bp and reads containing N ratios greater than 5% from the raw data. All downstream analyses were conducted based on the high-quality clean reads. The clean reads were mapped to the Sus scrofa genome (ftp://ftp.ncbi.nlm.nih.gov/genomes/all/GCF/000/003/025/GCF_000003025.5_Sscrofa10.2) using Tophat v2.0.9 with default parameters by calling bowtie2 [31,32]. The alignments were indexed and sorted with SAMtools for downstream convenience [33]. Transcripts were reconstructed using the Cufflinks v2.1.1 [31]. Read counts for gene expression were obtained using the HTSeq [34] software.

### 2.6. Identification of DEGs

Differentially expressed genes and corresponding adjusted *p*-values were determined using DEseq2 [35]. The significantly differentially expressed transcripts were declared at |log2(FoldChange)| > 1 and adjusted *p*-value < 0.01. Additionally, we used fragments per kilobase of transcript per million mapped reads (FPKM) as a measure that should be invariant to the length of genomic features in RNA-seq. FPKM were calculated using DEseq2 [35].

### 2.7. GO and KEGG Pathway Enrichment Analysis

The ClusterProfiler package, which is a new ontology-based tool, offers two methods (enrichGO, enrichKEGG) for gene enrichment analyses [36], was used to identify the main biological processes and metabolic pathways in differentially expressed genes (DEGs). For the gene ontology (GO) functional enrichment analysis, the parameters (organism, pig; ont, CC, MF, or BP; *p*-value cut-off, 0.05; P-adjust method, BH; readable, T) were used as the cut-off criteria. The GO enrichment results were visualized using the GOplot package [37]. For the Kyoto Encyclopedia of Genes and Genomes (KEGG) pathway enrichment analysis, the parameters (organism, pig; *p*-value cut-off, 0.05; *q*-value cut-off, 0.05; P-adjust method, BH; readable, T) were used as the cut-off criteria. The KEGG enrichment results were plotted using ggplot2 [38].

### 2.8. Reactome Pathway Enrichment Analysis

Reactome is a pathway database like KEGG. However, each database has biased and incomplete data. To more fully annotate the DEGs we obtained, we performed pathway enrichment of DEGs in the Reactome database. Since human genes have been better studied, all the pig gene IDs were converted to the IDs of homologous genes in humans. Then, the enrichment analysis was performed. The ReactomePA package was used to identify the main pathways in DEGs [39]. Default parameters (organism, human; *p*-value cut-off, 0.05; *q*-value cut-off, 0.05; minGSSize, 5; readable, T) were used as the cut-off criteria for Reactome pathway enrichment analysis.

### 2.9. PPI Network Construction

The Search Tool for the Retrieval of Interacting Genes (STRING) database was used to obtain PPI data. Similar to the Reactome database, the STRING database has more fully annotated human genes than pig genes. Therefore, we also converted the pig ID of the DEGs to the human ID before performing the analysis. The PPI networks of DEGs were visualized by Cytoscape (http://cytoscape.org/), which is an open source software for visualizing complex networks and integrating these networks with any type of attribute data [40].

### 2.10. Validation of Differentially-Expressed Genes by RT-qPCR

To evaluate the repeatability and reproducibility of gene expression data obtained by RNA sequencing, a real-time quantitative polymerase chain reaction (qPCR) assay using SYBR Green chemistry (SYBRTM Select Master Mix, Applied Biosystems) and the comparative Ct method was performed [41]. After thawing the purified RNA on ice, the isolated RNA from individual small intestine tissue samples was reverse-transcribed into cDNA using the PrimeScript RT Reagent Kit (TAKARA) in a total volume of 20 μL containing 1 μg of total RNA, according to manufacturer’s instructions. Real-time quantitative PCR was performed using Qiagen’s Quantitative Reaction Kit (QuantiFast SYBR Green PCR Kit, Qiagen, Hilden, Germany), 10 μL PCR system, with three replicates each. PCR parameters were: 95 °C denaturation, hot start 5 min; 45–50 PCR cycles (95 °C, 10 s, 60 °C, 15 s, 72 °C, 20 s); dissolution curve (95 °C, 15 s, 55 °C, 15 s, 95 °C, 15 s). All primers were designed using Oligo 7.0 software. Primers for quantitative PCR are shown in Table S6. The *ACTB* gene was used as an endogenous control.

### 2.11. Data Availability

The raw reads have been submitted to the NCBI Sequence Read Archive database (SRA) under BioProject accession number of PRJNA553821 and SRA accession number SRR9668622-SRR9668633.

## 3. Results

### 3.1. The Phenotype of the High and Low FE Groups Showed a Dramatic Difference

**Figure 1:**
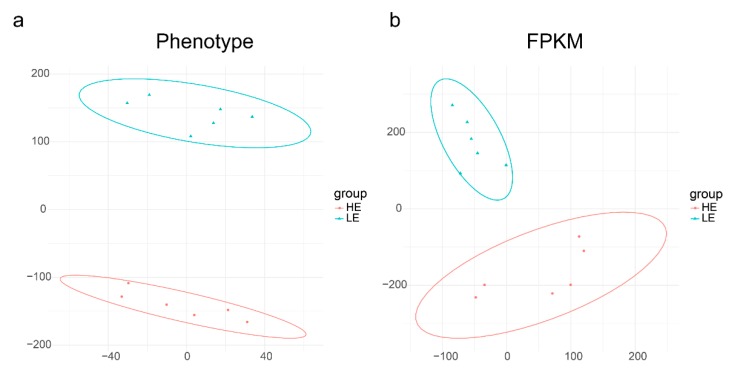
The t-distributed stochastic neighbor embedding (t-SNE) analysis of phenotype (**a**) and fragments per kilobase of transcript per million mapped reads (FPKM) (**b**) between HE- and LE-groups. (**a**). The t-SNE analysis performed on the feed efficiency (FE)-associated phenotype data. Dots represent samples and are colored according to FE phenotype: red (HE: high feed efficiency), blue (LE: low feed efficiency). The two group were well separated with six individuals in HE-group forming a cluster (red ellipse), and six individuals in LE-group forming a distinct cluster (blue ellipse). (**b**) The t-SNE analysis performed on FPKM expression values for all genes without a priori selection. Dots represent samples and are colored according to feed efficiency phenotype: red (HE: high feed efficiency), blue (LE: low feed efficiency). The two group were well separated with six individuals in HE-group forming a cluster (red ellipse), and six individuals in LE-group forming a distinct cluster (blue ellipse).

A total of 225 DLY sows were grown from 30–100 kg (average body weight) on a commercial pig feed (see Materials and Methods). Daily feed intake (DFI) and average daily gain (ADG) were measured and the FCR and RFI determined. From this data, two extreme groups were selected with either LE or HE. As expected, the HE-group had RFI and FCR values of −0.19 ± 0.08 (kg/day) and 2.17 ± 0.08, respectively, compared with 0.13 ± 0.1 and 2.68 ± 0.06 for the more efficient, LE-group (*p*-value = 7.19 × 10^−5^ and 8.91 × 10^−7^ for RFI and FCR, respectively; Table 1). RFI and FCR showed a strong positive covariation (phenotypic correlation r = 0.97; *p*-value = 2.50 × 10^−7^). The DFI of the HE-group was significantly lower than that of the LE-group (HE versus LE = 1.83 ± 0.15 versus 2.03 ± 0.13, *p*-value = 3.70 × 10^−2^). However, the ADG data in the 2 groups was the opposite of the DFI (HE versus LE = 0.85 ± 0.05 versus 0.77 ± 0.03, *p*-value = 1.14 × 10^−2^). Although the average metabolic body weight gain (AMBW) was higher in the HE-group, AMBW was not statistically significant (HE versus LE = 23.04 ± 0.24 versus 22.83 ± 0.21, *p*-value = 0.137). Due to the lower feed conversion rate, pigs in LE-group reached the final weight of 100 kg later. The testing days parameter of the LE-group was significantly longer (8.7 days) than the HE-group (HE versus LE = 82.17 ± 5.27 versus 90.83 ± 3.49, *p*-value = 8.85 × 10^−3^). The t-distributed stochastic neighbor embedding (t-SNE) analysis of the two groups phenotypic data clearly divides the individuals into two groups (Figure 1a). This result suggested strong correlations between the recorded phenotypes and that individuals within the group were highly reproducible.

### 3.2. Summary of RNA-seq Data

In this study, the RNA quality results are shown in Table S1 of the Supplementary Information; overall statistics of sequencing data we constructed from 12 cDNA libraries (six from HE small intestines, and six from LE small intestines). The sequencing generated, on average, 41,303,486 raw reads (20,651,743 paired reads) per sample. The raw reads were filtered and mapped to the Ss10.2 version of the pig genome (mapped rate, 79.89–82.78%). The analysis of the genes’ structure and distribution on the chromosomes of mapped reads are shown in Figure S1. Nearly 80% of read pairs mapped to exonic regions, 10% mapped to intronic regions, and the remaining 10% mapped to intergenic regions. The t-SNE analysis of the FPKM data of the two groups can clearly divide the individuals into two groups (Figure 1b). This result suggests that variations in gene expression patterns may cause phenotypic differences.

### 3.3. Differentially Expressed Genes between Low and High FE Pigs

**Figure 2:**
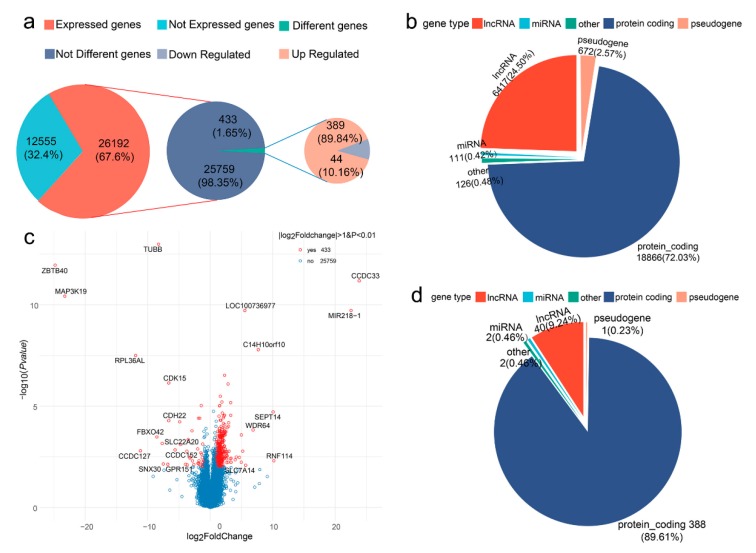
Counting and classification of differentially expressed genes (DEGs). (**a,b**) The pie chart displays the DEGs distribution among protein coding genes, long non-coding RNA (lncRNA), pseudogene, microRNA (miRNA), and other gene types. (**c**) Volcano plot of the DE genes with |log2(FoldChange)| > 1 and adjusted *p*-value < 0.01. The log2 Fold Change on the x-axis signifies expression difference, while the −log10 (*p*-value) on the y-axis indicates the significance of each gene. Red dots represent the 433 DE genes. Blue dots represent the remaining detected genes that did not meet the determined criteria. Dots with gene name annotations signify the 20 most significant DE genes. (**d**) The pie chart displays the distribution of 433 DE genes among protein coding genes, lncRNA, pseudogene, miRNA, and other gene types.

In the present RNA-seq study, 26,192 genes were detected in the small intestine of all 12 individuals (24,938 genes in the LE-group and 24,695 genes in the HE-group), accounting for 67.6% of the total annotated genes (Figure 2a). Most of these genes were protein-coding genes (72.03%), followed by long-chain noncoding genes (24.50%), pseudogenes (2.57%), and miRNAs (0.42%) (Figure 2b). A total of 433 differentially expressed genes (DEGs) with the criteria of |log2(FoldChange)| > 1 and adjusted *p*-value < 0.01 were identified (Supplementary Table S2). Of these 433 differentially expressed genes (DEGs), 389 genes were upregulated and 54 were downregulated in the HE-group (Figure 2a). The upregulated genes in the HE-group accounted for almost 90% of the DEGs, indicating that biological processes of some individuals in the HE-group may be more active. The proportion of protein-coding genes, long-chain noncoding genes, pseudogenes, and miRNAs in these DEGs were 89.61%, 9.24%, 0.23%, and 0.46%, respectively (Figure 2d). The top 20 differentially expressed genes (DEGs) are shown in Figure 2c; eight genes were upregulated while 12 genes were downregulated.

### 3.4. Functional Annotation Based on GO

**Figure 3:**
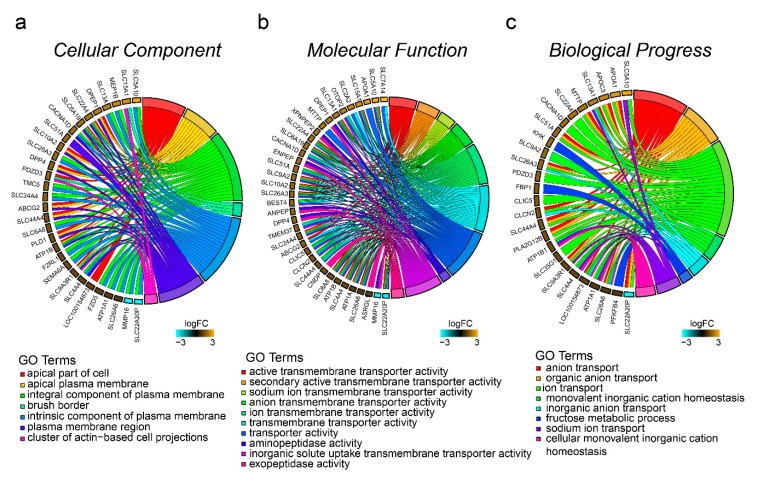
GOplot representation of the analysis of the gene ontology (GO) items enriched among the 433 differentially expressed genes. *p*-value, Benjamini-Hochberg-adjusted *p*-value. The left side of the circle displays the gene, and the right side shows the GO item. The assorted colors represent different GO items, and the color of each GO item is annotated below the circle. If the gene belongs to a GO item, there will be a line between the gene and the GO item. The z-score (bottom right) shows log2 (gene foldchange).

GO enrichment analysis results for the DEGs are shown in Figure 3. A total of 164 (37.88%) DEGs were annotated in the Gene Ontology database. GO analysis was performed for RNAs involved in cellular component (CC), molecular function (MF), and biological process (BP). The numbers of DEGs involved in these three categories were: 133 (30.72%), 135 (31.18%), and 120 (27.71%), respectively (Table S3). The genes enriched in these three classifications have a large overlap. In the CC category, there were two significantly enriched GO terms (Figure 3a)—the apical part of cell and the apical plasma membrane. Directed acyclic graphs (DAGs) are used to display the GO results. In these graphs, the relevance of the DE genes is illustrated by the branching pattern from top to bottom (Figure S2a). According to the DAG, the differential genes were mainly enriched in the composition of the cell membrane (Figure 3a and Figure S2a). Interestingly, all the differential genes enriched in the CC category were upregulated (Figure 3a), suggesting that cell membrane synthesis in the small intestine cells of the HE-group is more active. In the MF category, there were 43 significantly enriched GO terms (Table S3); the 10 most significant terms are shown in Figure 3b. The differential genes were mainly enriched in transporter activity, and the most significant term was active transmembrane transporter activity. According to the DAG, most of the differential genes were included in inorganic anion transmembrane transporter activity, solute:sodium symporter activity, ion antiporter activity, and inorganic solute uptake transmembrane transporter activity. There were 27 DEGs involved in the top 10 GO items, and all genes, except *SLC22A20P*, were upregulated (Figure 3b and Figure S2b). In the BP category, there were 16 significantly enriched GO terms (Table S3); the 10 most significant terms are shown in Figure 3c. These GO items were involved in single organism signaling, single-organism transport, and single-organism cellular process (Figure 3c). The most significant term was ion transport. According to the DAG, most differentially expressed genes were involved in organic anion transport, sodium ion transport, cofactor transport, monovalent inorganic cation homeostasis, and regulation of Ras protein signal transduction (Figure 3c and Figure S2c). There were two other signal transduction GO terms that had been enriched, Rho protein signal transduction and small GTPase-mediated signal transduction (Table S3).

### 3.5. KEGG Functional Enrichment Pathways

**Figure 4:**
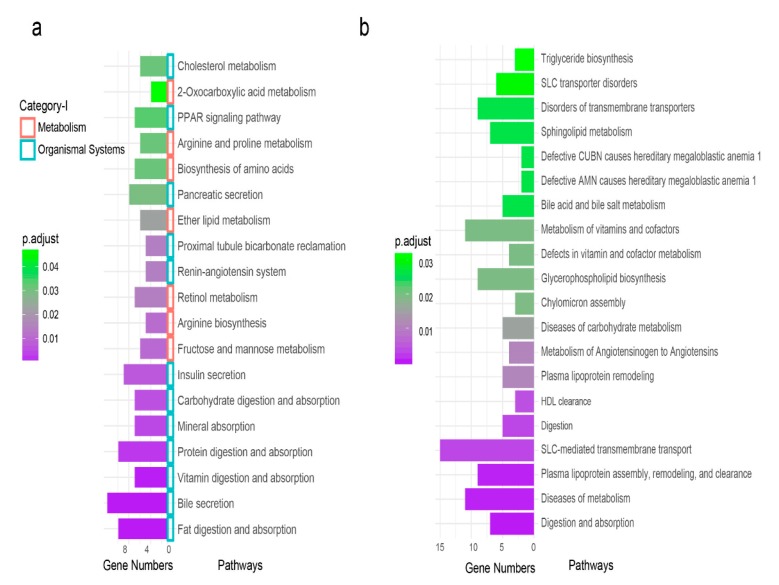
Pathway analyses of the DE genes. (**a**) Enriched pathways of the DE genes in the Kyoto Encyclopedia of Genes and Genomes (KEGG) pathway database. The vertical coordinates are the enriched pathways, and the horizontal coordinates are the number of DE genes in each enriched pathway. The color of the bar represents the adjusted *p*-value, while the small squares represent the pathway’s primary category. (**b**) Enriched pathways of the DE genes in the ReactomePA pathway database. The vertical coordinates are the enriched pathways, and the horizontal coordinates are the number of DE genes in each enriched pathway. The color of the bar represents the adjusted *p*-value.

Using the ClusterProfiler R package [36], we performed a Kyoto Encyclopedia of Genes and Genome (KEGG) [42,43,44] pathway enrichment analysis that links the genes observed within FE to relevant digestive system pathways. A total of 149 (34.41%) DEGs were annotated in the KEGG database, and 19 pathways were significantly (adjusted *p*-value < 0.05) enriched (Table S4). The pathways mainly fit into two primary categories, namely, metabolism (seven pathways) and organismal systems (12 pathways) (Figure 4a). These two categories also contained seven secondary classifications, including digestive system (eight pathways), endocrine system (three pathways), carbohydrate metabolism (one pathway), amino acid metabolism (two pathways), metabolism of cofactors and vitamins (one pathway), excretory system (one pathway), lipid metabolism (one pathway), and overview (two pathways) (Table S4). The enriched pathways covered the digestion and absorption of three major nutrients: fat, protein, and carbohydrates (Figure 4a). In addition, other pathways involving nutrient absorption, such as vitamin and mineral digestion and absorption, were included (Figure 4a). All DEGs enriched in these pathways were more highly expressed in the HE-group. Many of these genes largely overlapped with transporter activity genes from the GO enrichment analysis. This result indicated that the nutrient absorption in the HE-group was more active and effective.

The intestinal digestion and absorption require the coordination and regulation of other organs. Fortunately, two digestive fluid pathways (bile secretion and pancreatic secretion) and two regulation pathways (insulin secretion and peroxisome proliferators-activated receptor (PPAR) signaling pathway) were enriched. The DEGs in these four pathways were upregulated.

### 3.6. Reactome Functional Enrichment Pathways

In the Reactome database, 189 DEGs had annotation information, which accounts for 43.65% of all DEGs. We identified a total of 28 significant pathways (Table S5). The top 20 pathways are shown in Figure 4b. There were nine disease-related or dysfunctional disorders in our enrichment results. The remaining 19 were classified into three categories: (1) Digestion and absorption: digestion and absorption; solute carrier (SLC) -mediated transmembrane transport; digestion and intestinal absorption. (2) Catabolism: metabolism of angiotensinogen to angiotensin; metabolism of vitamins and cofactors; bile acid and bile salt metabolism; sphingolipid metabolism; fructose catabolism. (3) Fat transport and synthesis: plasma lipoprotein assembly, remodeling, and clearance; HDL clearance; plasma lipoprotein clearance; glycerophospholipid biosynthesis; triglyceride biosynthesis.

### 3.7. Protein-Protein Interaction (PPI) Network

**Figure 5:**
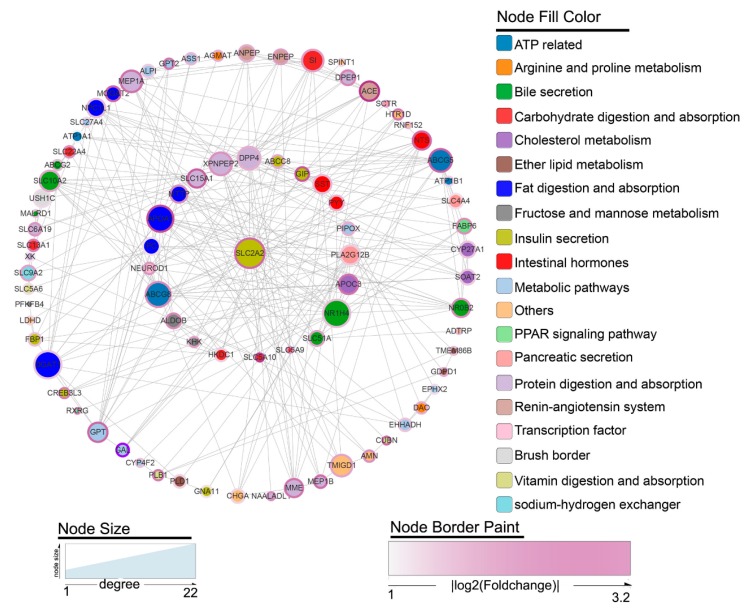
Protein-protein interaction (PPI) network constructed by the Search Tool for the Retrieval of Interacting Genes (STRING) database for DEGs. The two-level gene network relationship of the hub gene (*SLC2A2*) with the highest degree as the center. Node size indicates the degree of each gene. Node border color represents the fold change in gene expression. Node color represents the functional classification of the gene.

According to protein-protein interaction (PPI) network analysis, the two-level gene network relationship of the hub gene *SLC2A2*, which had the highest degree of relationship to other genes, was successfully constructed (Figure 5). *SLC2A2* showed the most protein interactions among the DEGs and 22 related genes were detected. Moreover, 61 indirect associations were identified through those 22 related genes. These 83 genes essentially covered the results of previous GO and pathway enrichment analyses (Figure 5).

### 3.8. Quantitative Real Time PCR Validation of Eight DEGs

**Figure 6:**
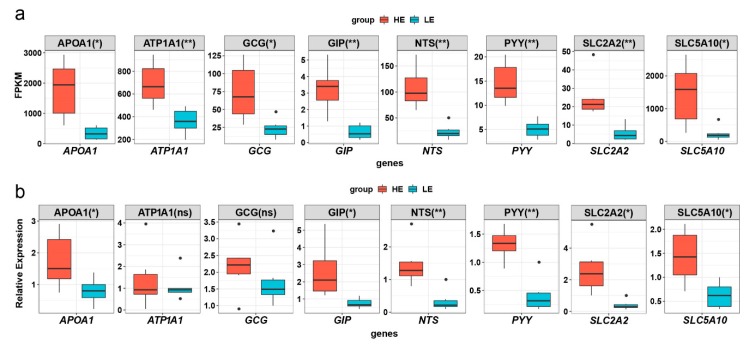
Quantitative real time polymerase chain reaction (qPCR) validation of eight DE genes. (**a**) Boxplot of FPKM expression values for the eight DE genes in RNA-seq. The y-axis represents the FPKM expression level, and the x-axis represents the gene name. The color of boxplot represents either the extremely high feed efficiency (HE)-group (red) or extremely low feed efficiency (LE)-group (blue). (**b**) Boxplot of the *APOA1*, *ATP1A1*, *GCG*, *GIP*, *NTS*, *PYY*, *SLC2A2*, and *SLC5A10* relative expression levels normalized to *ACTB*. * represents *p*-value < 0.05 in the HE-group compared with the LE-group. ** represents *p*-value < 0.01 in the HE-group compared with the LE-group.

To validate the DEGs, eight genes were selected for Quantitative real time PCR analysis. Among these genes, *APOA1*, *GIP*, *NTS*, *PYY*, *SLC2A2*, and *SLC5A10* reached a significant level of difference (*p*-value < 0.05) (Figure 6). Although *ATP1A1* and *GCG* did not attain significant levels in the results, the expression trends were consistent with the RNA-seq results (Figure 6).

## 4. Discussion

In this study, systematic transcriptome profiling of small intestine tissues from two groups of pigs with extreme opposing feed conversion efficiency was performed using high-throughput RNA-Seq technology. We found that the glucose sensitivity of the insulin pathway in small intestine mucosal tissues was important for FE in pigs.

In our study, feed efficiency was assessed using RFI and FCR. These two phenotypic traits showed a strong positive covariation, which is consistent with previous research [11]. The high feed efficiency directly caused pigs in the HE-group to reach 100 kg 8.7 days faster than the LE-group. This result is consistent with the findings in Yorkshire pigs [24]. These results suggest that improving feed efficiency not only saves feed costs but it also shortens the production cycle, which in turn reduces site costs and increases farming benefits.

GO analysis showed that DEGs are mainly enriched in the cell apical membrane composition and microvilli (brush border) composition of the cell composition classification. These data suggest that the number of small intestinal epithelial cells and microvilli area may be higher in the HE-group compared to LE-group. Thus, HE-group pigs may have greater absorption capacity because of their enhanced absorption structures. A previous study showed a 15% increase in the height of the villi in pigs with high feed efficiency [45]. In the molecular function and biological processes classification, the DEGs were mainly enriched in the entry of the transporter activity and transport process of fat, protein, and carbohydrates. Hence, the absorptive capacity of individual cells may also be higher in the HE-group and the absorption of nutrients may be more active. Glucose transporters were upregulated in Large White × Landrace pigs with high feed efficiency [46]. The variations between these entries may explain the differences in feed efficiency among individuals in the HE- and LE-groups.

We analyzed the enrichment pathways of DEGs, involving 19 pathways in the KEGG and 28 pathways in the Reactome databases. These enriched pathways focus on the digestion and absorption of various nutrients (e.g., fats, vitamins, proteins, and carbohydrates) as well as the regulation of hormone secretion (e.g., bile and insulin) in nutrient absorption. The results indicated that the HE-group’s ability to digest and absorb nutrients better than the LE-group may be a cause of the FE differences between the two groups.

Total absorption capacity can also increase by improving the absorption capacity per unit area, which is hormonally regulated. An important function of insulin is to stimulate the uptake of glucose from the intestines [47]. Through PPI network analysis of DEGs, we found a hub gene (*SLC2A2*), which plays an important role in the insulin secretion pathway. *SLC2A2*, also known as glucose transporter 2 (*GLUT2*), encodes a glucose transporter in the liver, intestine, and kidney epithelium [48]. Liang et al. found that *SLC2A2* gene expression was associated with pig growth rate and could affect fat deposition in pigs [49]. Furthermore, epidermal growth factor additives appeared to promote pig growth by increasing *SLC2A2* gene expression in the pig small intestine [50,51]. *SLC2A2* intestinal-knockout mice weighed less under the same feeding conditions [52]. In contrast, increasing *SLC2A2* expression in intestinal columnar epithelial cells enhanced glucose uptake [53]. Therefore, individuals in the HE-group with higher *SLC2A2* gene expression had greater ability to absorb glucose than the LE-group. *SLC2A2* also affects the HE in the HE-group in other ways. Double allele knockout of the mouse *SLC2A2* gene resulted in severe hyperglycemia, low insulin, and high glucagon in the blood [54]. When *SLC2A2* gene expression was repaired in the *SLC2A2*-/- mouse, glucose-stimulated insulin secretion returned to normal [55]. These mouse studies have shown that the *SLC2A2* gene mediates insulin secretion and regulates blood glucose balance as a glucose receptor [56]. Insulin can stimulate the uptake of glucose from the intestines [47]. Thus, we conclude that high *SLC2A2* expression in the HE-group results in greater glucose sensitivity and stimulates insulin secretion, which promotes glucose absorption and improves feed efficiency.

*SLC2A2* may also affect FE through other genes. Intestinal *SLC2A2* deletion in mice resulted in microvillus length reduction [52]. Hence, *SLC2A2* can regulate the growth of microvilli. *SLC2A2* is associated with insulin pathway genes, including *GCG* and *GIP*. *GCG* produces an important hormone, glucagon, which was significantly increased in the HE-group (*p*-value < 0.05). *GCG* is a preproprotein that is cleaved into glucagon-likepeptide-1 (*GLP-1*) and glucagon-likepeptide-2 (*GLP-2*). *GLP-1* is a potent stimulator of glucose-dependent insulin release [57]; it has growth-promoting activities on intestinal epithelium [58]. *GLP-2* has similar growth-promoting functions in the rat [59] and pig [60]. *GLP-1* and *-2* most likely play a role in promoting apical plasma membrane synthesis in the HE-group. *GLP-2* also enhances nutrient absorption by inhibiting gastric motility and secretion and stimulating nutrient transport [61]. *GIP* is an incretin hormone and belongs to the glucagon superfamily. The *GIP* protein is important for glucose homeostasis as it is a potent stimulator of insulin secretion from pancreatic β-cells following food ingestion and nutrient absorption. Previous studies also found higher levels of *GIP* expression in HE pigs [62]. According to current and previous studies, high *SLC2A2* expression may improve nutrient absorption capacity in the small intestine by regulating *GCG* and *GIP*.

There are no studies concerning *SLC2A2* or some of other DEGs (group of DEGs) as potential genes associated with feed efficiency at present. In future studies, researchers can identify molecular markers in these DEGs and their regulatory regions [63,64]. And these markers could be directly incorporated into artificial selection programs for increased feeding efficiency in pigs.

## 5. Conclusions

In this study, we performed RNA-seq analysis on small intestine mucosal tissues derived from 12 DLY pigs with either extremely high food efficiency (HE) or extremely low feed efficiency (LE). We found 433 significant DEGs between the HE- and LE-groups. Enrichment analysis revealed that the DEGs were mainly enriched in the apical plasma membrane composition, transporter activity, transport process, and hormone regulation of digestion and absorption. Protein network interaction and gene function analysis implied that *SLC2A2* was an important candidate gene for feed efficiency in pigs. Furthermore, some significant DEGs identified in the current study could be incorporated into artificial selection programs for increased feeding efficiency in pigs.

## Figures and Tables

**Table 1:** Performance of Duroc × (Landrace × Yorkshire) (DLY) pigs used in transcriptome sequencing.

Trait	HE (*n* = 6)	LE (*n* = 6)	*p*-value
SW (kg)	30.87 ± 1.54	30.13 ± 0.76	3.30 × 10^−1^
EW (kg)	100.23 ± 0.62	99.37 ± 1.17	1.48 × 10^−1^
TD (day)	82.17 ± 5.27	90.83 ± 3.49	8.85 × 10^−3^
ADG (kg/day)	0.85 ± 0.05	0.77 ± 0.03	1.14 × 10^−2^
DFI (kg)	1.83 ± 0.15	2.03 ± 0.13	3.70 × 10^−2^
FCR (kg/kg)	2.19 ± 0.08	2.68 ± 0.05	8.91 × 10^−7^
AMBW (kg)	23.04 ± 0.24	22.83 ± 0.21	1.37 × 10^−1^
RFI (kg/day)	−0.18 ± 0.08	0.14 ± 0.09	7.19 × 10^−5^

HE = high efficiency. LE = low efficiency. *n* = number of pigs. SW = starting weight. EW = ending weight. TD = testing days. ADG = average daily gain over the assessed feeding period. DFI = daily feed intake. FCR = feed conversion ratio. AMBW = average metabolic body weight gain. RFI = residual feed intake. *p*-value was calculated by *t*-test.

## Supplementary Materials

The following are available online at https://www.mdpi.com/2073-4425/10/9/725/s1, Figure S1: Alignment distribution of the uniquely mapped reads from RNA-seq in pig small intestine tissue, Figure S2: DAGs of the GO enrichment in DEGs, Table S1: Output statistics and annotation information of sequencing reads, Table S2: all DEGs, Table S3: Gene ontology enrichment result, Table S4: KEGG enrichment result, Table S5: ReactomePA pathway enrichment result, Table S6: Primers for quantitative real time polymerase chain reaction (qPCR).
