# Supplementary material for: A Transcriptome Analysis Identifies Biological Pathways and Candidate Genes for Feed Efficiency in DLY Pigs
Source: Genes (Basel). 2019 Sep 18;10(9):725. doi: 10.3390/genes10090725 (PMC6771153; doi:10.3390/genes10090725)
Supplement: Supplementary file 1 [file genes-10-00725-s001.zip › Supplementary files/Supplemental Table S1 Output statistics and annotation information of sequencing reads.docx]

Table S1. Output statistics and annotation information of sequencing reads.

| **Group** | **HE** | | | | | | **LE** | | | | | |
| --- | --- | --- | --- | --- | --- | --- | --- | --- | --- | --- | --- | --- |
| **Sample name** | **HE1** | **HE2** | **HE3** | **HE4** | **HE5** | **HE6** | **LE1** | **LE2** | **LE3** | **LE4** | **LE5** | **LE6** |
| Effective Reads | 41892794 (100%) | 40480632 (100%) | 40282090 (100%) | 40107530 (100%) | 41526726 (100%) | 41135020 (100%) | 41955822 (100%) | 41467316 (100%) | 41754330 (100%) | 41808506 (100%) | 41461212 (100%) | 41769864 (100%) |
| Total mapped | 34121046 (81.45%) | 32500721 (80.29%) | 32659981 (81.08%) | 33022122 (82.33%) | 33327131 (80.25%) | 33271629 (80.88%) | 34729262 (82.78%) | 33714056 (81.3%) | 33960815 (81.33%) | 33401949 (79.89%) | 33843163 (81.63%) | 34461722 (82.5%) |
| Multiple mapped | 1917647 (4.58%) | 1830732 (4.52%) | 1790680 (4.45%) | 1781385 (4.44%) | 1837491 (4.42%) | 1848911 (4.49%) | 1682874 (4.01%) | 1843155 (4.44%) | 1753983 (4.2%) | 1773622 (4.24%) | 1810860 (4.37%) | 1771402 (4.24%) |
| Uniquely mapped | 32203399 (76.87%) | 30669989 (75.76%) | 30869301 (76.63%) | 31240737 (77.89%) | 31489640 (75.83%) | 31422718 (76.39%) | 33046388 (78.76%) | 31870901 (76.86%) | 32206832 (77.13%) | 31628327 (75.65%) | 32032303 (77.26%) | 32690320 (78.26%) |
| Read1 mapped | 17409322 (41.56%) | 16595040 (41%) | 16629848 (41.28%) | 16734730 (41.72%) | 17290294 (41.64%) | 17228178 (41.88%) | 17618441 (41.99%) | 17134941 (41.32%) | 17273525 (41.37%) | 16899806 (40.42%) | 17204747 (41.5%) | 17461039 (41.8%) |
| Read2 mapped | 16711724 (39.89%) | 15905681 (39.29%) | 16030133 (39.79%) | 16287392 (40.61%) | 16036837 (38.62%) | 16043451 (39%) | 17110821 (40.78%) | 16579115 (39.98%) | 16687290 (39.97%) | 16502143 (39.47%) | 16638416 (40.13%) | 17000683 (40.7%) |
| Reads map to '+' | 17074311 (40.76%) | 16261923 (40.17%) | 16350121 (40.59%) | 16522737 (41.2%) | 16677743 (40.16%) | 16635541 (40.44%) | 17387792 (41.44%) | 16876144 (40.7%) | 16982812 (40.67%) | 16712234 (39.97%) | 16930183 (40.83%) | 17239592 (41.27%) |
| Reads map to '-' | 17046735 (40.69%) | 16238798 (40.11%) | 16309860 (40.49%) | 16499385 (41.14%) | 16649388 (40.09%) | 16636088 (40.44%) | 17341470 (41.33%) | 16837912 (40.61%) | 16978003 (40.66%) | 16689715 (39.92%) | 16912980 (40.79%) | 17222130 (41.23%) |
| Reads mapped in proper pairs(*2) | 15632128 (37.31%) | 14830078 (36.63%) | 15006051 (37.25%) | 15286972 (38.11%) | 15024098 (36.18%) | 15109910 (36.73%) | 16141259 (38.47%) | 15500068 (37.38%) | 15630850 (37.44%) | 15228196 (36.42%) | 15604624 (37.64%) | 15987928 (38.28%) |

Uses Sus scrofa 10.2 as the reference genome annotation to classify the mapping tags to the different regions. HE, high efficiency; LE, low efficiency.
